# Etherization in Puerperal Convulsions

**Published:** 1856-12

**Authors:** 


### *Etherization in Puerperal Convulsions.*

Dr. Griscom reports to the Philadelphia College of Physicians a case of puerperal convulsions occurring in a corpulent young woman, pregnant with her second child. She was suffering under general anasarcous swelling, and experienced vertigo occasionally. While dressing her hair, she fell down in violent apoplectic convulsions. Her pulse was full and rapid, and being unable to bleed her, Dr. G. took sixteen ounces of blood from her head by cups, and the same quantity by leeches. There was no evidence of labor having begun. As the patient still remained unconscious, and the convulsions were recurring with fearful violence, chloroform was used as a rubefacient to the spine and epigastrium, and ether given by inhalation. After its exhibition the convulsions became less and less violent, labor set in, and a living child was born. The mother recovered.—*Medical and Surgical Reporter.*

